# Midterm outcomes of aortic valve replacement using a rapid-deployment valve for aortic stenosis: TRANSFORM trial

**DOI:** 10.1016/j.xjon.2023.10.034

**Published:** 2023-11-17

**Authors:** S. Chris Malaisrie, Mubashir A. Mumtaz, Glenn R. Barnhart, Randolph Chitwood, William H. Ryan, Kevin D. Accola, Himanshu J. Patel, Y. Joseph Woo, Todd M. Dewey, Konstantinos Koulogiannis, Michael P. Dorsey, Eugene A. Grossi

**Affiliations:** a Division of Cardiac Surgery, Northwestern University, Chicago, Ill; b Department of Cardiovascular and Thoracic Surgery, University of Pittsburgh Medical Center Harrisburg, Harrisburg, Pa; c Structural Heart Program, Swedish Heart and Vascular Institute, Seattle, Wash; d Department of Cardiovascular Sciences, East Carolina University, Greenville, NC; e Cardiac Surgery Specialists, Baylor Plano Heart Hospital, Plano, Tex; f Florida Hospital Cardiovascular Institute, Florida Hospital Orlando, Orlando, Fla; g Department of Cardiac Surgery, University of Michigan, Ann Arbor, Mich; h Department of Cardiothoracic Surgery, Stanford University, Palo Alto, Calif; i Sarah Cannon Research Institute, Dallas, Tex; j Cardiovascular Core Lab at Morristown Medical Center, Morristown, NJ; k Department of Cardiothoracic Surgery, New York University Langone Medical Center, New York, NY

**Keywords:** aortic valve replacement, rapid deployment, sutureless valve, minimally invasive

## Abstract

**Background:**

The use of rapid-deployment valves (RDVs) has been shown to reduce the operative time for surgical aortic valve replacement (AVR). Long-term core laboratory–adjudicated data are scarce, however. Here we report final 7-year data on RDV use.

**Methods:**

TRANSFORM was a prospective, nonrandomized, multicenter, single-arm trial implanting a stented bovine pericardial valve with an incorporated balloon-expandable sealing frame. A prior published 1-year analysis included 839 patients from 29 centers. An additional 46 patients were enrolled and implanted, for a total of 885 patients. Annual clinical and core laboratory–adjudicated echocardiographic outcomes were collected through 8 years. Primary endpoints were structural valve deterioration (SVD), all-cause reintervention, all-cause valve explantation, and all-cause mortality. Secondary endpoints included hemodynamic performance assessed by echocardiography. The mean duration of follow-up was 5.0 ± 2.0 years.

**Results:**

The mean patient age was 73.3 ± 8.2 years. Isolated AVR was performed in 62.1% of the patients, and AVR with concomitant procedures was performed in 37.9%. Freedom from all-cause mortality at 7 years was 76.0% for isolated AVR and 68.2% for concomitant AVR. Freedom from SVD, all-cause reintervention, and valve explantation at 7 years was 97.5%, 95.7%, and 97.8%, respectively. The mean gradient and effective orifice area at 7 years were 11.1 ± 5.3 mm Hg and 1.6 ± 0.3 cm^2^, respectively. Paravalvular leak at 7 years was none/trace in 88.6% and mild in 11.4%. In patients undergoing isolated AVR, the cumulative probability of pacemaker implantation was 13.9% at 30 days, 15.5% at 1 year, and 21.8% at 7 years.

**Conclusions:**

AVR for aortic stenosis using an RDV is associated with low rates of late adverse events. This surgical pericardial tissue platform provides excellent and stable hemodynamic performance through 7 years.

## Graphical abstract

**Figure:**
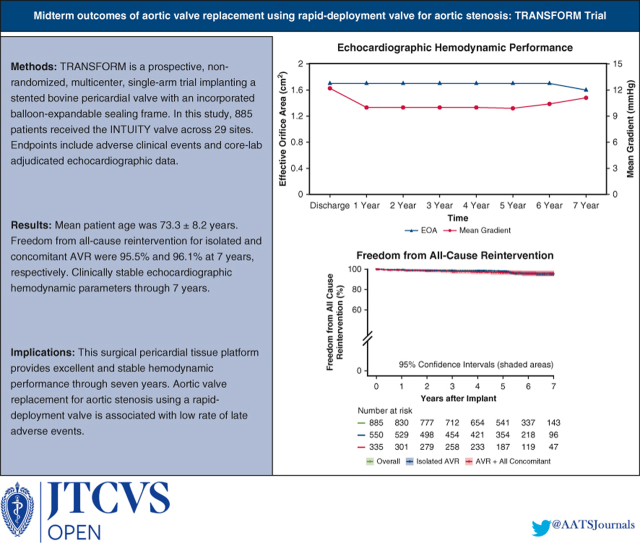

**Figure:**
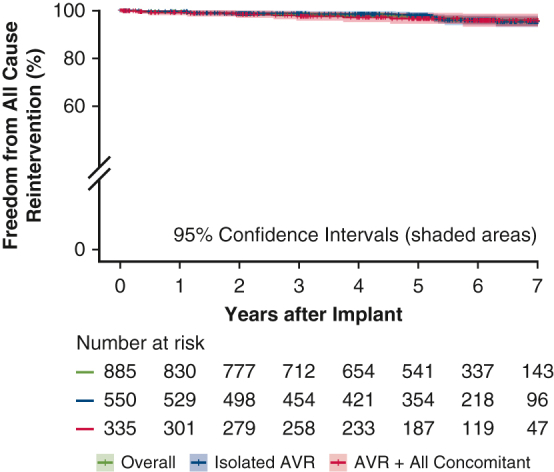
Freedom from all-cause reintervention at 7 years after rapid-deployment valve implantation.

**Central Message**

The INTUITY rapid-deployment valve provides excellent and stable hemodynamic results through 7 years, with a low rate of late adverse events.

**Perspective**

The use of bioprosthetic valves in aortic valve replacement demands scrutiny of durability over time. This multicenter clinical trial with core laboratory–adjudicated echocardiography followed 885 subjects receiving a rapid-deployment valve through 7 years, demonstrating restoration of survival to an age-matched cohort and durability comparable to contemporary surgical and transcatheter bioprosthetic valves.

Surgical aortic valve replacement (AVR) historically has been the standard for the treatment of patients with aortic stenosis. As techniques and technology have evolved, minimally invasive AVR has become an acceptable modality without an increased risk of major complications or death while offering faster recovery time, reduced blood loss, and shorter hospital length of stay.^1^ However, longer cross-clamp and bypass times associated with minimally invasive AVR have hindered its widespread use. Yet advancements in bioprosthetic valve design and the subsequent development of rapid-deployment valves (RDVs) has now changed this narrative. The use of RDVs has been shown to reduce operative times for surgical aortic valve replacement and facilitate minimally invasive approaches.^2^^,^^3^ At present, little prospective clinical long-term outcomes data are available for RDVs,^4^ and little core laboratory–adjudicated echocardiographic follow-up data are available for modern surgical bioprosthetic valves in general.^5^, ^6^, ^7^ AVR operations with concomitant procedures are within the domain of surgical AVR and technology to shorten cross-clamp times is tantamount in these complex patients. The self-anchoring technology of the INTUITY system facilitates rapid deployment, allowing for shorter cross-clamp times.

TRANSFORM was a medical investigational device study run under the authority of the US Food and Drug Administration (ClinicalTrials.gov NCT01700439). The purpose of the study was to assess the safety and effectiveness of the Edwards INTUITY Elite valve system (Edwards Lifesciences) in patients with aortic stenosis or stenosis-insufficiency necessitating primary replacement of the native aortic valve. This prospective, clinical, and core laboratory–adjudicated echocardiographic study evaluated a bioprosthetic valve surgically implanted with a rapid deployment technique with and without a minimally invasive approach.^8^^,^^9^ Here we present the final 7-year outcome of the INTUITY Elite valve system.

## Methods

The Edwards INTUITY Elite valve system is a rapid-deployment pericardial trileaflet stented aortic bioprosthesis. This design combines the technology of the Edwards Perimount Magna Ease (Edwards Lifesciences) bovine pericardial valve and a subannular balloon-expandable stainless steel cloth-covered frame that is deployed in the left ventricular outflow tract. A cloth skirt covers the balloon-expandable stainless steel inflow frame, providing a firm seal between the aortic annulus and the expanded frame. A prospective nonrandomized multicenter single-arm trial—TRANSFORM (Multicenter Experience with Rapid Deployment Edwards INTUITY Valve System for Aortic Valve Replacement; ClinicalTrials.gov NCT01700439)—was conducted to evaluate the performance of the aortic RDV in patients with severe aortic stenosis. The published 1-year analysis included 839 patients from 29 centers who underwent either isolated AVR or AVR with concomitant cardiac procedures.^3^ An additional 46 patients were enrolled and implanted, for a total of 885 patients from 2012 to 2016; these patients are the subject of this report.

**Figure 1:**
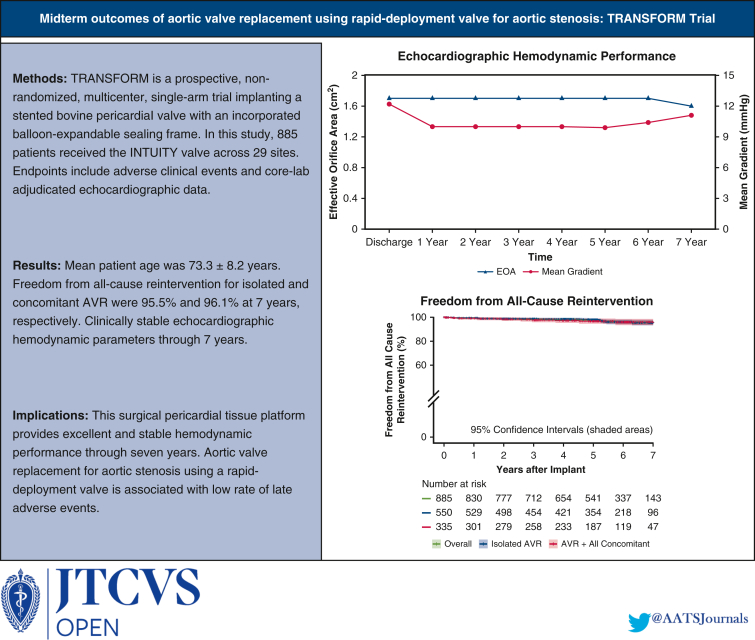
Graphical abstract. Results demonstrating echocardiographic hemodynamic performance and freedom from all-cause reintervention over 7 years. *EOA*, Effective orifice area; *AVR*, aortic valve replacement.

Study results were adjudicated by both annual clinical and core laboratory echocardiographic data through 8 years. Clinical endpoints as specified by Akins and colleagues' definitions were adverse events, including all-cause mortality, structural valve deterioration (SVD), all-cause reintervention, and all-cause explantation.^10^ Of note, reintervention also encompassed all percutaneous interventional catheter procedures. Hemodynamic performance was assessed by echocardiography. These endpoints are the basis for this report and are summarized in a graphical abstract (Figure 1). The mean duration of follow-up was 5.0 years. Follow-up compliance at 5 years was 90.7% of eligible patients for the visit, and 81.2% had a core laboratory echocardiographic evaluation. Survival of an age- and sex-matched cohort was constructed from the 2016 period life table for the Social Security population.^11^ TRANSFORM was conducted using Good Clinical Practices in accordance with 21 CFR Part 812 (Investigational Device Exemption), 21 CFR Part 50 (Protection of Human Subjects), CFR Part 54 (Financial Disclosure by Clinical Investigators), 21 CFR Part 56 (Institutional Review Boards), and the Health and Insurance Portability and Accountability Act. The clinical protocol, consent form, and protocol amendments were approved by an Institutional Review Board at each investigational site, as detailed in Table E1.

### Statistical Analysis

The sites were responsible for accurate data collection. Descriptive data including patient baseline characteristics and hemodynamic performance at baseline, 1 year, 5 years, and 7 years were summarized by mean with standard deviation for continuous variables and by frequency with percentage for categorical variables. Rates of freedom from SVD, all-cause reintervention, all-cause valve explantation, and all-cause mortality with 95% confidence intervals were estimated using Kaplan-Meier methods. All statistical analyses were performed using R version 4.2.3 (R Core Team) and SAS version 9.4 (SAS Institute).

## Results

**Figure 2:**
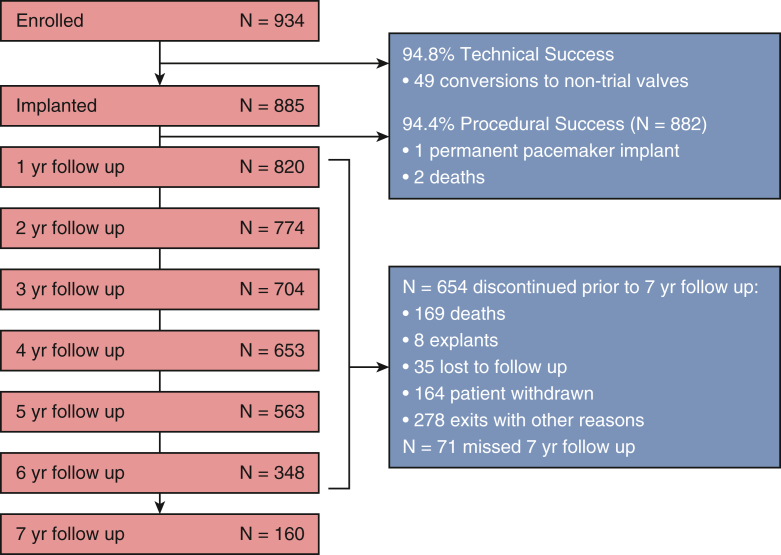
CONSORT diagram.

**Figure 3:**
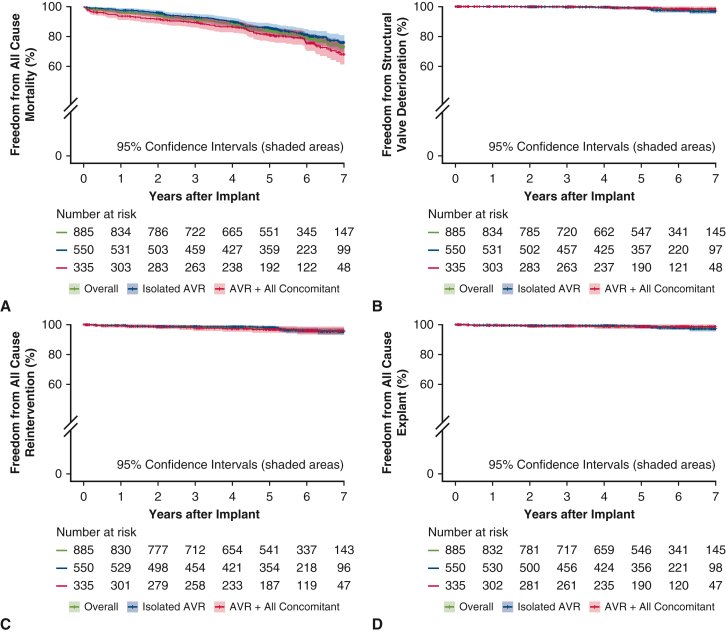
Kaplan-Meier estimates of freedom from all-cause deaths for all implanted patients (A), structural valve deterioration (B), all-cause reinterventions (C), and (D) all-cause explants (D). *AVR*, Aortic valve replacement.

**Figure 4:**
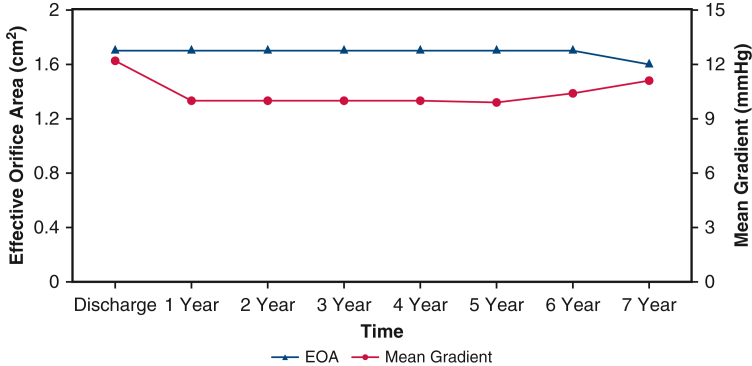
Echocardiographic hemodynamic performance over time. *EOA*, Effective orifice area.

**Figure E1:**
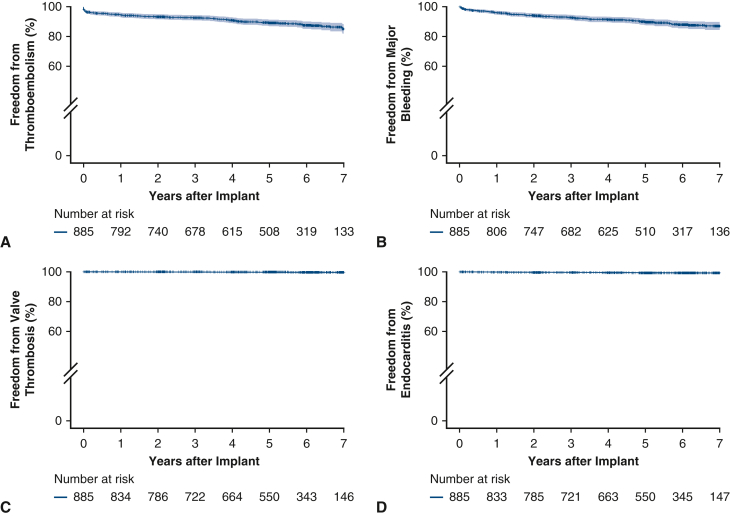
Kaplan-Meier estimates of freedom from thromboembolism (A), major bleeding (B), valve thrombosis (C), and endocarditis (D) for all implanted patients.

Figure 2 outlines the follow-up for the 885 implanted patients, and Table 1 presents their baseline demographics and operative variables. The mean patient age was 73.3 ± 8.2 years. Isolated AVR was performed in 62.1%, and AVR with concomitant procedures was performed in 37.9%. Table 2 outlines the perioperative 30-day outcomes as reported previously.^3^ Freedom from all-cause mortality at 7 years was 76.0% for isolated AVR and 68.2% for concomitant AVR (Figure 3, *A*). An age- and sex-matched cohort from the US general population had a predicted survival at 7 years of 72.3%. Freedom from SVD, all-cause reintervention, and all-cause explantation at 7 years were 97.5%, 95.7%, and 97.8%, respectively (Figure 3, *B-D*). A total of 13 explantations were performed in 7 years, in including 5 due to aortic regurgitation, 6 due to SVD, and 2 due to endocarditis. In addition, low rates of safety events were observed through 7 years, with rates of freedom from thromboembolism, major bleeding, valve thrombosis, and endocarditis of 85.2%, 87.2%, 99.6%, and 99.2%, respectively (Figure E1). Mean gradient and effective orifice area were 9.9 ± 5.5 mm Hg and 1.7 ± 0.3 cm^2^, respectively, at 5 years and 11.1 ± 5.3 mm Hg and 1.6 ± 0.3 cm^2^ at 7 years (Table 3, Figure 4). The degree of paravalvular leak (PVL) at 7 years was none/trace in 88.6% and mild in 11.4%. In the subset of patients undergoing isolated AVR, the cumulative probability of pacemaker implantation was 13.9% at 30 days, 15.5% at 1 year, and 21.8% at 7 years. Furthermore, the 7-year cumulative probability of pacemaker implantation for complete heart block was 10.9%.

**Table 1:** Baseline characteristics and operative variables of implanted subjects (N = 885)

Baseline characteristics/operative variables	Value
Age, y, mean ± SD (range)	73.3 ± 8.2 (34.0-95.0)
Sex, % (n)	
Female	35.3 (312)
Male	64.7 (573)
NYHA classification (N = 882), % (n)	
Class I	15.3 (135)
Class II	53.1 (468)
Class III	29.9 (264)
Class IV	1.7 (15)
Risk scores, mean ± SD (range)	
STS risk of mortality, %	2.5 ± 1.8 (0.4-14.6)
Euroscore II, %	3.3 ± 3.3 (0.5-31.6)
Surgical procedure, % (n)	
Isolated AVR	62.1 (550)
AVR + CABG	25.2 (223)
AVR + CABG + other	4.1 (36)
AVR + other	8.6 (76)
Cross-clamp time, min, mean ± SD	68.8 ± 36.9
Cardiopulmonary bypass time, min, mean ± SD	
Isolated AVR, full sternotomy	69.2_± 34.7
Isolated AVR, MIS	84.6 ± 33.5
AVR + CABG 1 grafts	87.1 ± 34.2
AVR + CABG 2 grafts	113.3 ± 38.4
AVR + CABG 3 grafts	140.6_± 44.0
AVR + CABG 4 grafts	171.0 ± 44.4

*SD*, Standard deviation; *NYHA*, New York Heart Association; *STS*, Society of Thoracic Surgeons; *AVR*, aortic valve replacement; *CABG*, coronary artery bypass grafting; *MIS*, minimally invasive surgery.

**Table 2:** Perioperative outcomes reported previously (N = 839)

30-d outcome	Value
All-cause mortality, % (n)	0.8 (7)
Valve-related mortality, % (n)	0.5 (4)
Reoperation, % (n)	0.2 (2)
Valve explant, % (n)	0.1 (1)
Stroke, % (n)	2.6 (22)
Valve thrombosis, % (n)	0 (0)

**Table 3:** Hemodynamic outcomes: Echocardiography-derived mean gradients

Visit	Mean gradient, mm Hg, n; mean ± SD; range	EOA, cm^2^, n, mean ± SD; range	DVI, n; mean ± SD; range	Vpeak Ao, m/sec, n; mean ± SD; range
Baseline	866; 43.7 ± 15.05; 10.2-105.9	849; 0.7 ± 0.19; 0.3-1.8	855; 0.2 ± 0.04; 0.1-0.4	866; 4.2 ± 0.70; 2.2-6.5
1 y	787; 10.0 ± 3.56; 3.1-28.7	766; 1.7 ± 0.32; 1.0-2.9	768; 0.4 ± 0.04; 0.2-0.7	787; 2.1 ± 0.36; 1.2-3.5
5 y	479; 9.9 ± 5.46; 3.2-60.5	454; 1.7 ± 0.32; 0.7-2.5	459; 0.4 ± 0.18; 0.2-4.1	479; 2.1 ± 0.45; 1.2-5.1
7 y	101; 11.1 ± 5.28; 4.2-38.2	95; 1.6 ± 0.32; 0.6-2.4	96; 0.4 ± 0.05; 0.1-0.5	101; 2.2 ± 0.47; 1.4-4.3

*SD*, Standard deviation; *EOA*, effective orifice area; *DVI*, Doppler velocity index; *Vpeak Ao*, aortic peak velocity.

## Discussion

**Figure 5:**
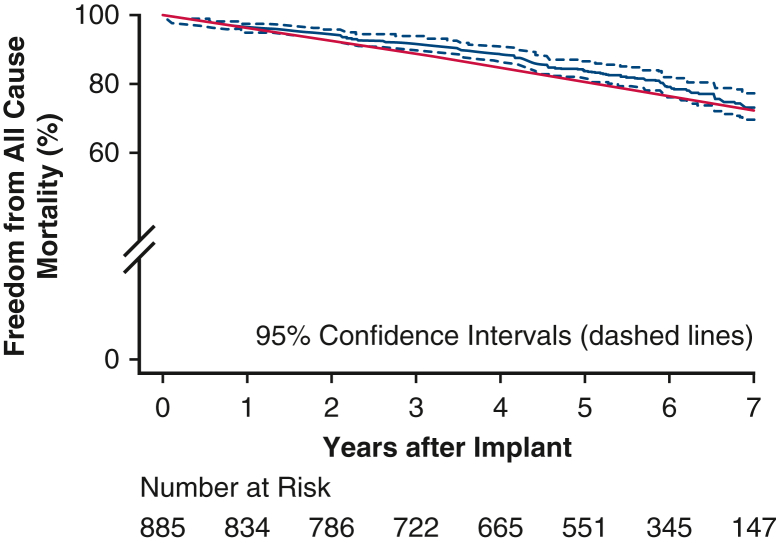
Freedom from all-cause mortality comparing the TRANSFORM cohort (*blue line*) and the age/sex-matched SSA cohort (*green line*). *SSA*. Social Security Administration.

The TRANSFORM trial is one of the largest US Investigational Device Exemption trials of a contemporary bioprosthetic valve. A previous study of 1-year results using this class of bioprosthetic valves demonstrated both safety and efficacy.^3^ This current analysis based on 885 patients found rates of overall survival, freedom from SVD, and freedom from all-cause reintervention at 7 years of 73.2%, 97.5%, and 95.7%, respectively. No patients had moderate or greater PVL, and the mean gradient was 11.1 mm Hg at 7 years. Previously noted pacemaker implantation rates^12^ were stable, at a cumulative rate of 21.8% for isolated AVR at 7 years. Additionally, 7-year overall survival after AVR with the INTUITY valve was similar to that in an age- and sex-matched US population (73.2% vs 72.3%) (Figure 5).

Although our results are early, the data reinforce the previously reported 8-year outcomes for the Magna Ease valve, which offers promise for continued durability.^13^ Leaflet treatment aims to reduce the calcification of free aldehydes that results from the fixation process of biological tissue. The proprietary process is unique to each manufacturer; in this particular valve, the ThermoFix process was used for all implanted INTUITY valves. As such, acceptable gradients are stable after 7 years and compare favorably with earlier generations of this bioprosthetic valve (Table 2, Figure 3, *B*).

Although no patients had moderate or greater PVL, the rate of mild PVL was 11.4% at 7 years. In comparison, the rates of mild PVL associated with other surgical valves include 1.6% at 8 years for Magna Ease^13^ and 0.9% at 5 years for Avalus.^6^ Additionally, moderate-severe PVL at 5 years for current generation of transcatheter heart valves is 21.6% (Notion trial).^14^ The importance of mild PVL as a marker for the future progression of leak, risk of endocarditis, and hemolysis has been debated but has not been borne out in this study. For transcatheter AVR patients, even mild paravalvular insufficiency at 1 month postprocedure has been identified as a marker for increased 5-year mortality.^15^^,^^16^

Regarding permanent pacemaker implantation (PPI), Romano and colleagues^12^ reported an all-cause PPI rate of 12.3% at discharge and 15.1% at 1 year after AVR in all patients. The all-cause PPI rate in patients without baseline conduction abnormalities was 6.6% at discharge and 9.2% at 1 year. An independent predictor of PPI was a baseline right bundle branch block (odds ratio, 7.35; *P* < .0001). Interestingly, subset analysis revealed a >2-fold difference in observed PPI rates among the top 4 enrolling institutions, which may be a reflection of those institutions' different discharge strategies, given the median time to PPI before discharge of 5 days. This may reinforce the guideline of waiting 5 days to see whether permanent pacing is required and/or necessary. After commercialization of the INTUITY device, several institutions have shown a lower rate of PPM with increased experience and enhanced technique.^17^^,^^18^ A follow-up study revealed only a single new PPI in 87 patients at risk over the 2 years of monitoring, for a cumulative rate of 10.1%.^18^ This is in alignment with the reported 2-year cumulative PPI rates of 9.1% for transcatheter AVR and 7.0% for surgical AVR in the PARTNER 3 study.^19^

### Limitations

There are limitations to this study. First, the trial was single-armed, and thus no comparative effectiveness analysis could be done with control valves that were available at the time of trial enrollment. Second, rehospitalization rate was not a secondary endpoint. At the time of trial design, valve endpoints were guided by the Akins guidelines, as Valve Academic Research Consortium endpoints were not available. We supplemented for this by including transcatheter aortic valve-in-valve or PVL closure as reinterventions. Finally, no further follow-up is planned in the TRANSFORM trial.

## Conclusions

AVR for aortic stenosis using a RDV is associated with a low rate of late adverse events. Our analysis of 7-year follow-up data from the TRANSFORM prospective nonrandomized multicenter single-arm trial using the Edwards INTUITY Elite valve system shows that the valve system has consistently demonstrated sustained and comparable clinical and hemodynamic outcomes. This dataset provides a contemporary baseline performance benchmark for surgical AVR for aortic stenosis.

## Conflict of Interest Statement

Dr Malaisrie reported research grants, consulting fees, and honoraria from 10.13039/100006520Edwards Lifesciences, 10.13039/100004374Medtronic, Artivion, and Terumo Aortic. Dr Mumtaz reported consulting fees, honoraria, and research grants from 10.13039/100006520Edwards Lifesciences, 10.13039/100004374Medtronic, 10.13039/100007330AtriCure, 10.13039/100024655Teleflex, and 10.13039/100000046Abbott. Dr Barnhart reported consulting for Articure and Artivion. Dr Chitwood reported consulting for NeoChord and Edwards Lifesciences. Dr Accola reported consulting fees and honoraria from Edwards Lifesciences. Dr Dewey reported serving as a speaker for Abbott as well as a speaker, proctor, and consultant for Edwards Lifesciences. Dr Koulogiannis reported consulting for Edwards Lifesciences and Abbott. Dr Grossi reported intellectual property and royalties from Medtronic for valve repair devices and intellectual property and royalties from Edwards Lifesciences. Drs Ryan, Patel, Woo, and Dorsey reported no conflicts of interest.

The *Journal* policy requires editors and reviewers to disclose conflicts of interest and to decline handling or reviewing manuscripts for which they may have a conflict of interest. The editors and reviewers of this article have no conflicts of interest.

## Figures and Tables

**Table E1:** IRB approvals

Site name	IRB name	City, state	Approval no.	Date of initial approval
Cleveland Clinic Foundation	Cleveland Clinic IRB	Cleveland, OH	12-1344	29 Jan 2013
New York University	NYU School of Medicine IRB	New York, NY	S12-03267	20 Jan 2013
Hoag Memorial Hospital	Western Institutional Review Board (WCG)	Newport Beach, CA	20121284	19 Mar 2013
Stanford University	Stanford University IRB	Palo Alto, CA	348	6 May 2014
University of Michigan	University of Michigan Medical School IRB	Ann Arbor, MI	HUM00066926	1 Nov 2012
Advent Health System/Florida Hospital Cardiovascular Institute	Florida Hospital IRB	Orlando, FL	318730-4	26 Sep 2012
Duke University	Duke Medicine IRB	Durham, NC	Pro00043677	20 Jun 2013
Washington University/Barnes-Jewish Hospital	Washington University in St Louis Human Research Protection Office	St. Louis, MO	201208134	26 Sep 2012
Aurora St. Luke's Medical Center	Aurora Health Care IRB	Milwaukee, WI	13-38	27 Mar 2013
Columbia University Medical Center	Columbia University Medical Center IRB	New York, NY	IRB-AAAO2706	8 Oct 2014
University of California Los Angeles	University of California Los Angeles IRB	Los Angeles, CA	12-001278	6 Feb 2013
Mission Hospital	Mission Health System IRB	Asheville, NC	13-01-970, F	31 May 2013
University of Pennsylvania	University of Pennsylvania IRB	Philadelphia, PA	816629	7 Nov 2012
University of Pittsburgh Medical Center	Western Institutional Review Board (WCG)	Pittsburgh, PA	20121284	5 Apr 2013
Swedish Health Services	Western Institutional Review Board (WCG)	Seattle, WA	20121284	10 Aug 2012
Northwestern University	Northwestern University IRB	Chicago, IL	STU00068982	20 Nov 2012
St. Thomas Health	Sterling IRB	Nashville, TN	4540-001	5 Nov 2013
Medical City Dallas	North Texas IRB at Medical City	Dallas, TX	2012.048	6 Sep 2012
Pinnacle Health Cardiovascular Institute	Pinnacle Health Hospitals IRB	Harrisburg, PA	PHH13-034	13 Aug 2013
East Carolina University	East Carolina University IRB	Greenville, NC	12-001405	26 Sep 2012
Dignity Health (Mercy General Hospital)	Sacramento-Sierra Regional IRB	Sacramento, CA	SAC-2013-076	19 Sep 2013
University of Southern California	University of Southern California Health Sciences Campus IRB	Los Angeles, CA	HS-13-00609	8 Nov 2013
North Shore Long Island Jewish Health System	Biomedical Research Alliance of New York IRB	Manhasset, NY	12-02-441-03	4 Feb 2013
The Cooper Health System	The Cooper Health System IRB	Camden, NJ	RP# 12-196	2 May 2013
Baptist Memorial Hospital Memphis	Baptist Memorial Hospital IRB	Memphis, TN	13-30	23 Sep 2013
The Heart Hospital Baylor Plano	Baylor Research Institute IRB	Plano, TX	012-179	16 Sep 2012
Good Samaritan Hospital	TriHealth IRB	Cincinnati, OH	12099-12-060	12 Oct 2012
Beth Israel Deaconess Medical Center	Beth Israel Deaconess Medical Center IRB	Boston, MA	2012-P-000422/1; BIDMC	19 Jun 2013
Baylor College of Medicine	IRB for Baylor College of Medicine and Affiliated Hospitals	Houston, TX	H-33047	20 Dec 2013

*IRB*, Institutional Review Board.
